# The Stress of Caring—Resilience and HPA-Axis Activity in Hair Samples of Youth Residential Caregivers

**DOI:** 10.3389/fpsyt.2020.556486

**Published:** 2020-12-21

**Authors:** David Bürgin, Nina Kind, Martin Schröder, Vera Clemens, Jörg M. Fegert, Anne Eckert, Anna Buchheim, Aoife O'Donovan, Cyril Boonmann, Marc Schmid

**Affiliations:** ^1^Child and Adolescent Psychiatric Research Department, University Psychiatric Hospitals, University of Basel, Basel, Switzerland; ^2^Department for Child and Adolescent Psychiatry and Psychotherapy, University Hospital Ulm, Ulm, Germany; ^3^Neurobiological Laboratory for Brain Aging and Mental Health, Transfaculty Research Platform, University of Basel, Basel, Switzerland; ^4^Institute of Psychology, University Innsbruck, Innsbruck, Austria; ^5^Department of Psychiatry and Weill Institute for Neurosciences, University of California, San Francisco, CA, United States; ^6^Mental Health Services, San Francisco Veterans Affairs Medical Center, San Francisco, CA, United States

**Keywords:** attachment, HPA axis, DHEA, cortisol, self-care, self-efficacy, sense of coherence, resilience

## Abstract

**Background:** Professional caregivers in youth residential care institutions experience frequent verbal and physical aggression as well as multiple stressors as part of their everyday work, leading to high levels of burnout and staff turnover. Resilience might buffer against psychophysiological stress response and therefore be crucial for well-being in professional caregivers.

**Objectives:** We aimed to investigate if measures related to resilience [sense of coherence (SoC), self-efficacy and self-care] and attachment security of caregivers were cross-sectionally associated with stress markers in hair samples [cortisol and dehydroepiandrosterone (DHEA)].

**Method:** Participants (*n* = 134; 64.2% women) reported on individual resilience measures and provided hair samples for cortisol and DHEA assays. Attachment was assessed in a subsample using the Adult Attachment Projective Picture System (AAP, *n* = 69). Linear regression models were fitted to estimate the association between resilience measures and the Cortisol:DHEA ratio, cortisol and DHEA, controlling for gender and age.

**Results:** SoC was associated with a lower Cortisol:DHEA ratio (β = −0.36, *p* < 0.001), driven by a positive association between SoC and DHEA levels (β = 0.28, *p* = 0.002). Self-care was also associated with lower Cortisol:DHEA ratios (β = −0.24, *p* = 0.005), due to self-care being associated with higher DHEA (β = 0.21, *p* = 0.016). HPA-axis measures were not associated with self-efficacy nor with attachment patterns in a subsample.

**Conclusions:** Our findings imply that youth residential care institutions might benefit from programs focusing on enhancing SoC and self-care practices. Fostering a meaningful, comprehensible and manageable professional climate in caregiving environments and implementing self-care in routine practices might enhance not only well-being but also physical health of professional caregivers and in this way buffer adverse health effects of chronic stressors.

## Introduction

Professional caregivers in child welfare institutions experience multiple stressors, including frequent episodes of verbal and physical aggression and are faced with complex mental health issues of often highly traumatized clients as part of their everyday work, leading to high levels of burnout, staff turnover, and compassion fatigue (1–3). Chronic stressors are well known to alter stress responses, impair immune function and accelerate aging processes (4–6). Resilience might buffer against individual psychophysiological stress responses and could therefore be an important contributor to enhanced well-being in professional caregivers. Therefore, this paper aims to investigate if individual resilience measures [sense of coherence (SoC), self-efficacy and self-care] and attachment security of caregivers were associated with stress hormone markers in hair samples [Cortisol, dehydroepiandrosterone (DHEA), and their ratio].

Professional caregivers in youth residential care are exposed to chronic stressors and aggression resulting from their work with a highly troubled clientele. Most children and adolescents in the youth welfare system grew up in highly disrupted families, were exposed to multiple traumatic stressors or were victims of child maltreatment, and thus have an increased risk to develop mental health problems such as anxiety, depression, externalizing disorders, substance abuse, and suicidal ideation (7–19). Beyond the impact on mental health, adverse and traumatic experiences might lead to long-lasting impacts on the development of one's self-concept and identity development, inhibitory control, relationships and attachment to others, including caregivers (20–24). Therefore, it is not surprising that professional caregivers in youth welfare institutions are at increased risk to develop burnout and secondary traumatic stress symptoms or might be prone to frequent change of workplaces and compassion fatigue (3, 25, 26). Trauma exposures in juveniles may further increase the risk of hostility and physical violence against caregivers (27, 28), which in turn are related to burnout risk and psychophysiological stress responses (2). In light of the considerable stress and trauma histories of children and adolescents within child welfare systems and the high risk for chronic stress and physical and verbal aggression in their caregivers, health and well-being of professional caregivers is of essence to ensure high quality of continuous care.

Resilience is a broad and multifactorial phenomenon and concept, which comprises the ability to “bounce back” in the face of adversity and chronic stressors but also includes personal growth after adverse experiences (29, 30). With the steadily increasing attention to research on resilience in the last decades, a lively and interdisciplinary discussion about what constitutes resilience emerged (31, 32). Important resilience factors include: at least one healthy attachment figure, good caregiving and parent-child interactions, emotion regulation abilities, self-awareness and future orientation, as well as mastery, perceived social support, sense of coherence and self-efficacy (31–34). Beyond psychological perspectives on resilience, biological components of resilience include genetic, epigenetic, and psychophysiological factors (6, 35, 36). Improving and fostering resilience might thus be a promising way to enhance well-being, work-satisfaction, and health of professional caregivers to ensure a high quality of care for such a highly troubled clientele.

Concepts commonly investigated within the scope of resilience of professional caregivers in the child welfare system are sense of coherence (SoC), self-efficacy and self-care, with attachment security among caregivers becoming of increasing interest.

- The concept of SoC was established as an integral variable of the concept of salutogenesis related to the professional and healthy functioning of an individual. It contains three domains of one's perception of life as being: comprehensible, manageable and meaningful (37, 38). Antonovsky proposed SoC and general resistance resources (GRR) as two main components of salutogenesis (37). GRR can include characteristics of an individual, a group or an environment (genetic, physical, and psychosocial), which might ease effective tension management (38). Numerous studies have shown SoC to be positively related to health-behaviors and mental health and negatively toward burnout, depression, anxiety and PTSD symptoms in caregivers and medical staff (1, 26, 39–41). Others showed SoC to be an important moderator and mediator between chronic stressors, early adversity and later health outcomes (42).
- Perceived self-efficacy is conceptualized as people's core beliefs in their ability to influence events that shape their lives, which thus is the foundation of their motivation, performance accomplishments, and subsequently their emotional well-being (43, 44). A meta-analyses of 57 studies showed self-efficacy being related to lower levels of work-related burnout (45). In foster parents and child welfare staff, self-efficacy was found to be related to continued caregiving and work/caregiving satisfaction (46, 47).
- Self-caring behavior in our study, in contrast to SoC and self-efficacy, is a more pragmatic way of measuring specific health-fostering behaviors in youth residential caregivers. Self-care contains physical factors (e.g., participating in sports, sleeping enough, balancing nutrition), psychological factors (e.g., feeling supported, upholding values, self-reflection) and work-related factors (e.g., taking breaks, successfully transitioning from work to private life, sharing responsibilities). Self-care was previously shown to be related to lower levels of burnout and lower levels of compassion fatigue (25, 26, 40, 48).
- Beyond these commonly studied constructs, attachment security was shown to be related to higher burnout and compassion fatigue levels in a review on studies in health and human service workers, as well as in dementia caregivers (49, 50).

These concepts are promising targets to enhance well-being, job satisfaction and professional functioning working in such a high stress job environment. Beyond these psychological outcomes of chronic stressors, psychophysiological measures of stress responses are of utmost interest to direct and target preventive measures toward those that also enhance physical health.

One possible way to measure the body's biological stress response is to measure hormones of the hypothalamic-pituitary-adrenal (HPA) axis, which plays a key role in responses to acute and chronic stressors. The HPA-axis is activated by the release of corticotropin-releasing hormone (CRH) in the hypothalamus, followed by the release of adrenocorticotropic hormone (ACTH) in the anterior pituitary. ACTH then initiates the synthesis and release of cortisol and dehydroepiandrosterone (DHEA) in the adrenals (51). Both cortisol and DHEA enable effective stress responses via the regulation of basal processes, such as for example immune responses and inflammatory processes (51–53). Chronically high cortisol is known to promote psychiatric illness in part through neurotoxic effects (51, 54–56), whereas DHEA is supposed to have neuroprotective effects potentially related to inhibitory effects on cortisol, due to the support of neurogenesis, and its antioxidant and anti-inflammatory effects (51, 54, 57). Measuring cortisol in saliva and blood allows assessment of acute stress responses, while obtaining cortisol and DHEA levels in hair samples allows assessment of longer-term stress responses. In particular, hair samples allow assessment of the accumulation of cortisol over time (5, 58). A commonly used measure to assess both stress hormones simultaneously is the ratio between cortisol and DHEA, which represents the balance between these two stress hormones (51, 59).

Work on chronic stress and severe early stress showed the HPA-axis to be associated with both increased or decreased stress system activity (60), with an initial hypercortisolism followed by a downregulation of the system (52). Findings on lower levels of basal DHEA in the context of chronic stress and a higher Cortisol:DHEA ratio might thus be understood as indication for a shift toward cortisol at the expense of DHEA, which would first manifest as a downregulation of DHEA, followed by a downregulation of cortisol leading to higher Cortisol:DHEA ratios (51, 61).

Despite the importance of understanding factors associated with resilience in residential youth caregivers, no study has yet investigated the association of resilience measures with cortisol and DHEA in hair samples in such a highly stressed population. Even though DHEA is a promising marker with neuroprotective potential, most studies focus on cortisol and less on DHEA mostly measuring real-time fluctuations in blood and saliva. The first studies assessing hair cortisol and DHEA to date either investigated small samples of traumatized adolescents or healthy adults (62–68). To our knowledge, this is the first study investigating the association between resilience and stress markers in hair samples in caregivers. Our study aims to investigate if individual resilience measures and attachment security of caregivers are associated with stress markers in hair samples. Specifically, we investigated the association of SoC, self-efficacy and self-care on hair cortisol, DHEA and the Cortisol:DHEA ratio. In addition, we assessed differences in hair cortisol, DHEA and the Cortisol:DHEA ratio between professional caregivers with different levels of attachment security in a subsample.

## Materials and Methods

### Procedures and Sample

Surveys and well-established questionnaires were mailed to participating institutions (fourteen institutions accredited by the Swiss Ministry of Justice) at four annual sampling points. Data on sociodemographic variables, experiences of private and work-related stressors, and self-caring behavior were collected using the surveys. SoC and perceived self-efficacy were based on questionnaires. Additionally, strands of hair (3 cm) adjacent to the scalp were sampled from the posterior vertex region. Given a general growth rate of 1 cm/month, cumulative cortisol and DHEA exposures over the last 12 weeks were assumed to be indexed (55). All participants provided written informed consent. The leading Ethics Committee “Basel-Stadt” and “Basel-Land” (EKBB, Ref. Nr. 288/12), as well as the Cantonal Ethics Committee Bern (KEK-BE, Ref. Nr. 014/13), Ethics Committee St. Gallen (EKSG, Ref. Nr. 13/003), Ethics Committee Appenzell Ausserrhoden (EKAR, Ref. Nr. 34), Cantonal Ethics Committee Luzern (KEK-LU, Ref. Nr. 13009) and the Cantonal Ethics Committee Zürich (KEK-ZH, Ref. Nr. 2013-0030) approved the overall project.

A total of 164 youth residential caregivers were enrolled in the larger overall project. However, 30 of those did not provide at least one useable hair samples or data on variables for main analyses on at least one measurement timepoint during the study. In a previous analysis using cortisol data, we showed that 80% of missing data were due to participants either refusing to provide hair samples or mostly due to hair being too short to provide samples (3 cm), therefore two third of missings were men and slightly older as those participating. Included participants however did not differ on other psychosocial variables (2). Thus, 134 caregivers (48 men, 86 women) were included in the current study that provided at least one hair-sample during the course of the study (descriptives are reported in the Result section). Additionally, a subset of 69 participants additionally took part in a projective method to assess attachment style during the course of the study. These data were then used to conduct cross-sectional analyses.

### Measures

#### Psychosocial Measures

##### Sense of coherence

The SoC in regard to daily work was assessed with a well-established German short version of the “Sense of Coherence Scale” by Antonovsky (7-point Likert scale with 9 items, scored 1 to 7) (69, 70). The mean was reported in the analyses. The authors of the German version reported Cronbach's alpha of 0.87 (70).

##### Self-efficacy

The perceived self-efficacy of caregivers was assessed with a well-established questionnaire developed for teacher populations and slightly adapted by the authors for professional caregivers (4-point Likert scale with 10 items, scored 1 to 4, from 1 = “not true,” 2 = “hardly true,” 3 = “rather true,” 4 = “exactly true”) (71). The mean score of all items is reported in the analyses. The authors reported Cronbach's alphas between 0.71 and 0.92 (71, 72).

##### Self-care

This self-developed questionnaire assessed physical, psychological and work-related self-caring behavior (73). The reference period reflected the past 3 months (4-point Likert scale, 24 items, scored from 1 to 4, 1 = “not accurate,” 2 = “rather not accurate,” 3 = “rather accurate,” 4 = “entirely accurate”). After conducting a principal components analysis to reduce data, three factors were extracted and rotated using promax-rotation (kappa = 4): (a) physical factors (e.g., participating in sports, sleeping enough, balancing nutrition), (b) psychological factors (e.g., feeling supported, upholding values, self-reflection) and (c) work-related factors (e.g., taking breaks, successfully transitioning from work to private life, sharing responsibilities). In our sample, Cronbach's alpha was 0.84. The selectivity of the items ranged from 0.22 to 0.59, while item difficulty ranged from 0.56 to 0.93. The total score mean was calculated for further analyses.

##### Adult attachment projective picture system (AAP)

The AAP is a widely used free response measure to assess adults' attachment representation. By using a set of standardized questions, individuals are asked to tell a story in response to one neutral and seven attachment-related picture stimuli depicting scenes of solitude, death, separation and fear designed to activate the attachment system in different settings (74–77). In the present study AAP stories of the participants were recorded, transcribed, and coded by a certified judge. Story coding reflects evaluation of story content (agency of self, connectedness, and synchrony), defensive processes (deactivation, cognitive disconnection and segregated systems) and the inclusion of personal experience (76). Attachment research works with a concept of a continuum from attachment security to attachment insecurity. In a recent study a rhombus structure for the AAP was suggested operationalized as an attachment security scale by considering the classification “secure” to be on the highest position, the classification disorganized classification U (“unresolved trauma”) on the lowest position and the two remaining organized insecure classifications “dismissing” and “preoccupied” on positions between secure and unresolved (78). In this present study we used this structure for measuring different levels of attachment security.

### Hair Cortisol and DHEA

Hair was collected from the posterior vertex region of the scalp. Due to variability on lengths of strands of hair only strands of hair (1.5 cm long) adjacent to the scalp were analyzed. Hair cortisol and DHEA were extracted in line with the protocol by Gao et al. (79). Cortisol levels were determined using a commercially available high-sensitivity (analytical sensitivity 0.007 μg/dL) cortisol enzyme immunoassay kit (Salimetrics Europe, UK) and DHEA levels using a Salivary DHEA ELISA kit (Salimetrics Europe, UK) according to the manufacturer's protocols. Evaporated samples were resuspended in assay diluent provided by the manufacturer. The intra-assay and inter-assay coefficients of variation of these assays are below 9%. Samples were analyzed in duplicate, and mean values of respective concentrations were calculated in pg/mg hair and used in statistical analyses. All measures were performed in blinded fashion.

### Analytic Plan

**Figure 1:**
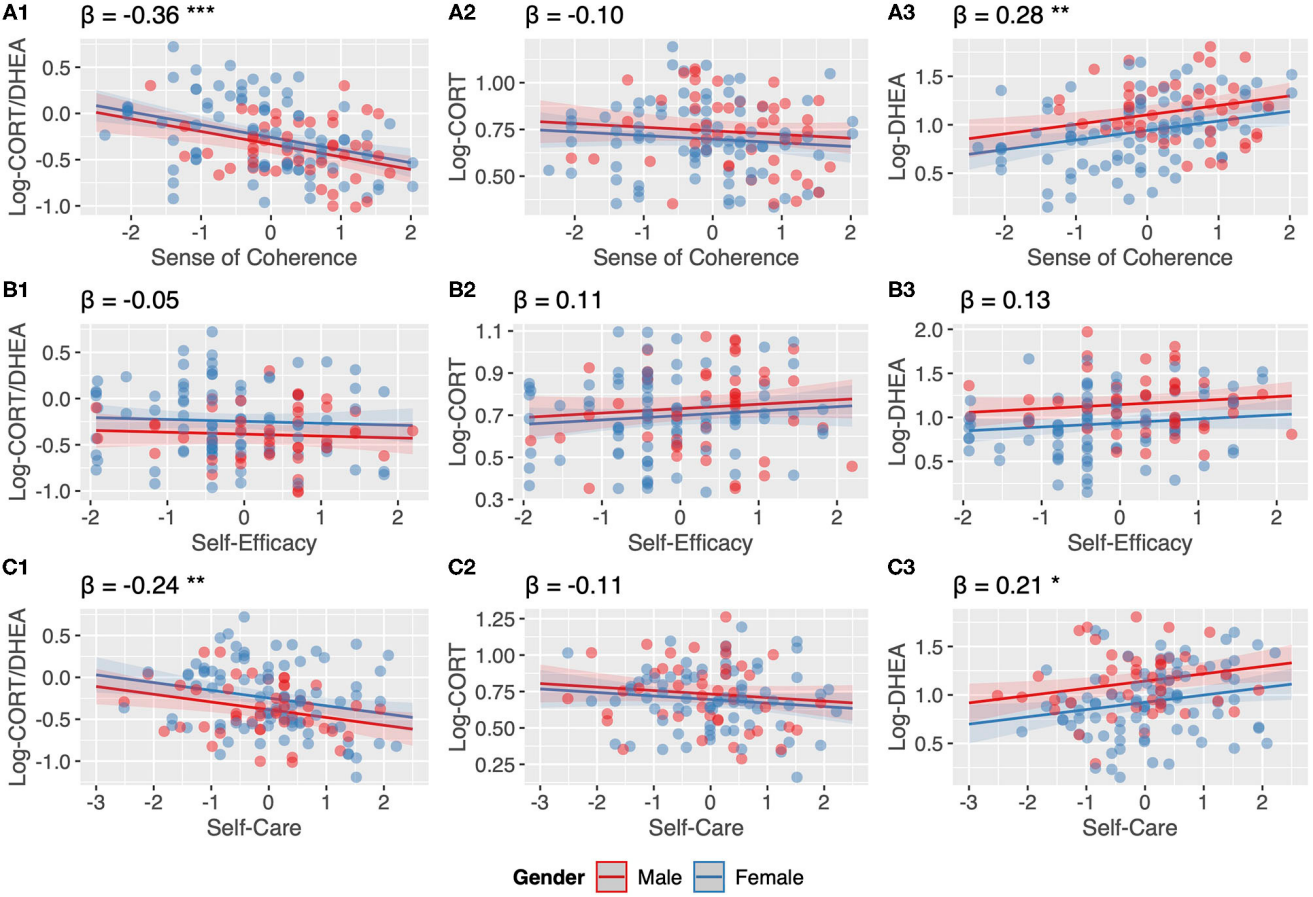
**(A1–C3)** Associations between stress measures in hair samples and resilience measures from linear regression models including age and gender as covariates. β = standardized regression coefficients, * *p* < 0.05 ** *p* < 0.01 *** *p* < 0.001.

Before data analyses, all variables were analyzed to assess their distribution and to handle outliers. Because the distributions of cortisol, DHEA and the Cortisol:DHEA ratio were skewed these variables were log-transformed with the common logarithm (log10), which has been recommended for hormone ratios (80). After log-transformation, data were normally distributed. Univariate outliers were defined as values below the 2.5% and over the 97.5% percentile. These values were capped onto the 2.5 and 97.5% percentile for the log-transformed Cortisol, DHEA, and the Cortisol:DHEA ratio, as well as for the SoC, self-efficacy and self-care scales. These last three scales were then z-transformed to standardize these measures. Multivariate outliers were assessed for each regression model separately by calculating the Cook's Distance and excluding cases with values larger than 5 times the mean from each of the models (displayed in Figure 1), leading to slightly different sample sizes for regression models (*n* = 130–134, indicated for each of the models, see Supplementary Tables).

**Figure 2:**
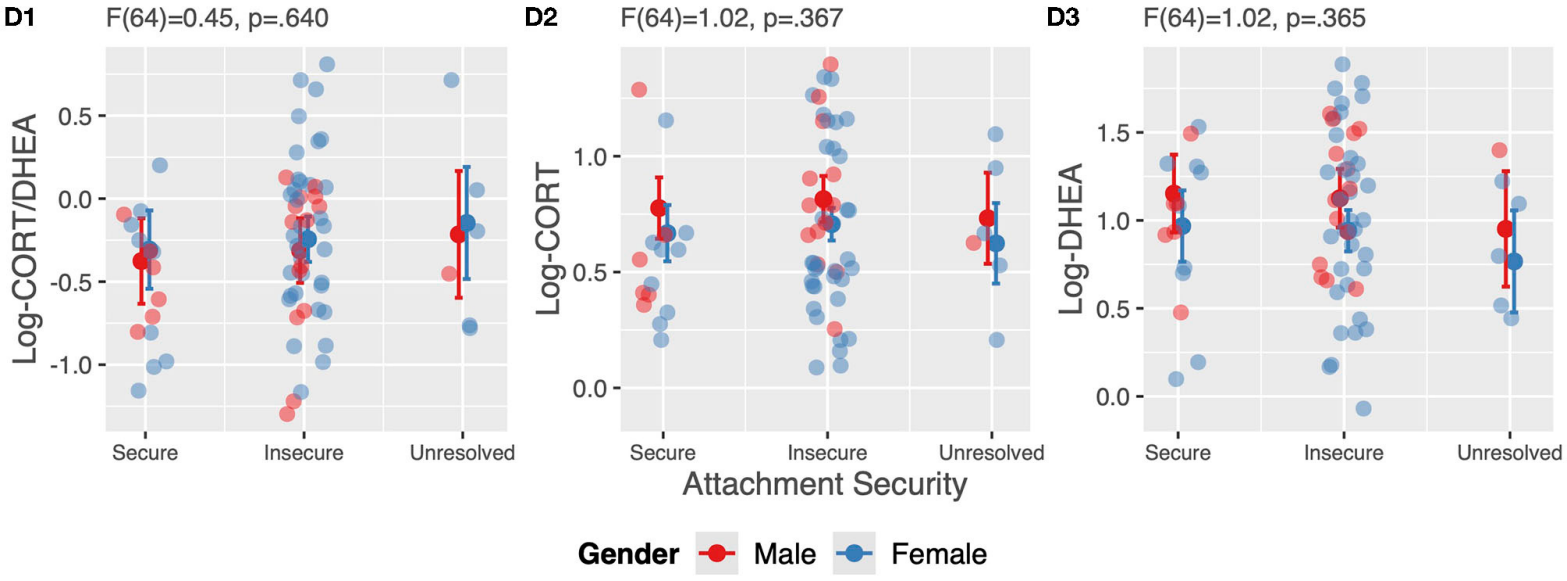
ANOVA assessing differences in stress levels in hair samples between professional caregivers with different attachment representations including age and gender as covariates.

All analyses in this study are cross-sectional in nature. Analyzing the data, we first assessed the association between sociodemographic variables and the Cortisol:DHEA ratio, cortisol and DHEA using univariate analyses of variance (ANOVA) and Spearman's correlations (see Table 1). Second, we assessed the correlation between the Cortisol:DHEA ratio, cortisol and DHEA using Pearson's correlations (see Table 2). Third, we fitted linear regression models to assess the association between resilience measures (SoC, self-efficacy and self-care) and stress measures (Cortisol:DHEA, cortisol and DHEA) including age and gender in each of these models. Results are reported in Figure 1 (plot A1–C3, additional data of these models are reported in the Supplementary Materials). We then conducted gender sensitivity analyses by including a gender x resilience interaction term in our models. Results are reported in Supplementary Figure 1 (plot A1–C3 and additional data of model A3 are reported in the Supplementary Materials). Last, we assessed differences in stress measures in hair samples of professional caregivers with different attachment representations in a subsample using ANOVA including age and gender in these models (see Figure 2). The statistical software used was R through RStudio (Version 3.5.2, 2018), Boston, MA, USA (81). Plots were created using the R-packages: “sjPlot” package (82) and “ggplot2” package (83). *P*-values for all models are indicated at the levels *p* < 0.05, *p* < 0.01, *p* < 0.001.

**Table 1:** Analysis of sociodemographic variables and stress measures from hair samples.

	**Log-Cort/DHEA**		**Log-Cort**		**Log-DHEA**	
	**M (SD)**	***p***	**M (SD)**	***p***	**M (SD)**	***p***
**Gender**^a^						
Male (*N* = 48)	−0.40 (0.35)	0.033^*^	0.75 (0.21)	0.156	1.14 (0.35)	0.002^**^
Female (*N* = 86)	−0.25 (0.41)		0.69 (0.21)		0.94 (0.36)	
**Stable relationship**^a^						
Yes (*N* = 97)	0.29 (0.40)	0.989	0.72 (0.20)	0.832	1.01 (0.37)	0.965
No (*N* =27)	−0.29 (0.41)		0.73 (0.25)		1.00 (0.39)	
**Own children**^a^						
No (*N* = 85)	−0.26 (0.40)	0.061	0.72 (0.20)	0.375	0.97 (0.37)	0.106
Yes (*N* = 49)	−0.39 (0.37)		0.69 (0.23)		1.08 (0.339)	
	r	*p*	r	*p*	r	*p*
Age^b^	−0.1	0.246	−0.1	0.229	0.08	0.331
Current empl. (yrs.)^b^	0.01	0.905	0.04	0.616	0.02	0.836
Work exp. (yrs.)^b^	0.03	0.736	0.01	0.868	−0.01	0.894
Work stressors ^b^	0.12	0.151	0.12	0.177	−0.05	0.555
Personal stressors^b^	−0.11	0.191	−0.02	0.855	0.09	0.28

*M, mean; SD, standard deviation; r, correlation coefficient*.

a *ANOVA*.

b *Spearman's correlations*.

* p < 0.05

** p < 0.01

*** *p < 0.001*.

**Table 2:** Pearson's correlations between Log-Cort/DHEA, Log-Cort and Log-DHEA.

	**Log-Cort**		**Log-DHEA**	
	***r***	***p***	***r***	***p***
Log-Cort/DHEA	0.37	<0.001^***^	−0.84	<0.001^***^
Log-Cort	–		0.19	0.032^*^

*r, correlation coefficient*.

* p < 0.05

** p < 0.01

*** *p < 0.001*.

## Results

### Descriptives

In total, 134 professional caregivers were included in the subsequent analyses. Of all participants 35.8% were men, 64.2% were women. Participants had a mean age of 35.20 years (SD = 9.54, range = 22–61) and were recruited from 14 residential youth welfare institutions. On average, they had 7.7 years (range = 0–37) of working experience in residential youth welfare institutions and had worked in the respective institution for a mean of 2.85 years (range = 0–18). Analyses of the association between sociodemographic variables with Cortisol:DHEA ratio, cortisol, and DHEA revealed gender-differences with women showing significantly higher Cortisol:DHEA ratio scores, due to significantly lower DHEA values in women compared to men (see Table 1).

All other associations between sociodemographic variables and stress measures and hair samples did not reach significant levels (see Table 1). Cortisol and DHEA levels were significantly, low to moderately correlated to each other, as were cortisol and DHEA values to the Cortisol:DHEA ratio (see Table 2).

### Associations Between Resilience and Stress Measures

SoC was found to be negatively associated (β = −0.36*)* with the Cortisol:DHEA ratio, mainly due to a positive association between SoC and DHEA (β = 0.28) (see Figures 1A1,A3). Self-efficacy was not found to be associated with the Cortisol:DHEA ratio, nor with Cortisol and DHEA (see Figures 1B1,B3). Self-care was negatively associated with the Cortisol:DHEA ratio (β = −0.24), largely to due to its positive association with DHEA (β = 0.21) (see Figure 1C1,C3). More detailed information on these regression models (R^2^, CIs, and exact *p*-values) are provided within Supplementary Material.

### Gender-Sensitivity Analyses

All models shown in Figure 1 were rerun including a gender x resilience interaction term. We found a gender-interactions in only one of these models, namely in regard to SoC and Log-DHEA (model A3). The positive association between SoC and DHEA is only found for women and not for men (β*-interaction* = 0.18). Plots regarding all of these models and a table regarding model A3 are to be found within the Supplementary Material.

### Association of Attachment Security and Stress Measures

A subsample of 69 caregivers provided additional data on attachment security. Overall, 21.7% (*N* = 15) of caregivers were classified as secure, 69.6% (*N* = 48) as insecure (dismissing and preoccupied) and 8.7% (*N* = 6) as unresolved. We found no group differences among attachment representation groups in Cortisol:DHEA ratios, nor in levels of cortisol and DHEA (see Figure 2).

## Discussion

In this cross-sectional study on HPA axis measures in hair samples of 134 youth residential caregivers, we found resilience factors (SoC and self-care) to be negatively associated with participants' Cortisol:DHEA ratio, due to their positive association with DHEA. Secondary analyses in a subsample of participants showed no group differences in psychophysiological stress measures between groups of caregivers with different attachment styles. These findings add to only a few studies investigating the associations of resilience with cortisol and DHEA and is the first to use hair samples, a rather new approach to measure chronic HPA-activation.

Our main finding was that resilience is negatively associated with the Cortisol:DHEA ratio in hair samples due to positive associations with DHEA. In addition, we found that the association between SoC and DHEA was moderated by gender, the positive association between SoC and DHEA are only found in women. This is in line with findings of a study in 32 non-clinical adults that indicated resilience to be related to lower Cortisol:DHEA ratio in hair samples, due to a significant positive association with DHEA-S (64), a sulfate of DHEA, which in its function is closely related to its predecessor (54). In a study of female adolescents in the West Bank, sense of family coherence was shown to be moderately negatively associated with the hair Cortisol:DHEA ratio in traumatized participants only and positively associated with hair DHEA only in participants with PTSD (65). However, another study found no association between SoC and any of the biomarkers (65). A pilot-study of 40 healthy participants investigating hair samples found no significant associations between SoC and a resilience scale with DHEA nor with cortisol (66). In line with this study, we did not find significant associations of any of our resilience measures with cortisol in independent models including one resilience factor at a time (see Figure 1). Two other studies, however, found negative associations between resilience and cortisol (84, 85). Taken together, to date only a small amount of studies with small samples of limited age ranges, different assessment methods of cortisol and DHEA, and often female only either traumatized adolescents or healthy adults were published in regard to resilience and DHEA or Cortisol:DHEA ratios. Our findings add to this heterogenous body of research and provide first evidence of resilience factors being negatively associated with the Cortisol:DHEA ratio in a sample of chronically stressed adults working as youth residential caregivers.

In secondary analyses, we did not find differences in psychophysiological stress measures based on different levels of attachment security of caregivers. To our knowledge, this is the first study that assessed differences in hair cortisol, DHEA and their ratio due to different attachment representations. Our findings might be influenced by the fact, that we only had 69 participants and groups sizes with respect to attachment were not balanced (Ns = 15, 48, 6), therefore our models might be underpowered. We used the AAP, an interview using a set of ambiguous attachment related pictures representing scenes of solitude, death, separation and fear by asking the participants to tell stories to these pictures. Since youth residential caregivers are highly exposed to many extreme and horrifying stories of abuse and deprivation of their clients, these experiences from the professional context might have been projected into these attachment pictures leading to a higher degree of attachment insecurity than expected. In our sample the distribution of attachment representations was more comparable to clinical rather than healthy samples (86). This might be one explanation why no differences with respect to psychophysiological levels were found. In contrast to our results, a study in 40 healthy college students, found subjective experiences of attachment-based overprotective parental rearing to be associated with higher DHEA and lower Cortisol:DHEA ratios (87). Taken together, our results should be considered as preliminary findings on attachment security in caregivers and their association with psychosocial stress measures in hair samples. In a recent study, we found attachment-related adverse childhood experiences, in particular maternal mental illness and frequent change of caregivers to be associated with higher Cortisol:DHEA ratios in a sample of children, adolescents and young adults living in residential youth care (88). Attachment security might be an important moderator between stressors and health outcomes, as was shown between childhood adversity and cellular aging (89). Considering attachment security in future research might account for some of the heterogeneity found in the association between (early) stressors and cellular aging across studies (4, 90). Thus, more research in particular on attachment security in high stress environments and high-risk samples is warranted.

### Limitations

Despite our reasonably large sample of youth residential caregivers and multiple measures of resilience analyzed in primary analyses, our findings need to be seen in light of some important limitations. First, our analyses are cross-sectional and therefore causality cannot be implied, and findings should be interpreted in light of this limitation. Second, reports on resilience measures were solely based on self-reports and recall and other biases might be apparent. Third, we were only able to measure cortisol and DHEA in the first 1.5 cm of hair and therefore only the last 6 weeks of cortisol and DHEA secretion (5), other studies, however, were able to assay 3 cm. Assessing only 1.5 cm however enabled us to include more men, whereas other studies only investigate women for this reason. Fourth, our secondary analyses of differences in psychophysiological stress measures between groups of different attachment style are to be considered only preliminary in nature due to the rather small sample size especially when comparing groups. Moreover, we realized that assessing the AAP with caregivers in this professional trauma context has to be taken in caution and special training for administration has to be ensured in order to minimize confounds and in consequence an oblique distribution. Fifth, as we had no information on other confounding factors such as diabetes, alcohol, body-mass-index, or high blood pressure that were found to influence hair cortisol (5), we were only able to control for gender and age. Sixth, we were not able to control for different work conditions within specific institutions, due to the small sample size on the institution level. Last, resilience as a broad and multifactorial phenomenon with to date no gold-standard of measurement is hard to grasp properly from a theoretical perspective, which is beyond the scope of this paper. Therefore, we used three concepts (SoC, self-efficacy, and self-care) as proxies for overall resilience. SoC and self-efficacy are well and broadly studied concept, whereas for self-care to date there is no clear and homogenous construct defined in the literature and it is very heterogeneously assessed. These concepts were found to be moderately correlated with one another (40), which complicates individual interpretations if combined in the same model, therefore we decided to analyze them separately.

### Implications

In light of our findings, promoting and enhancing resilience in caregivers might counteract the well-known adverse psychophysiological alterations associated with chronic stress (4, 5, 51). These cross-sectional correlational findings might be a first indicator of this possible health promoting effect and should be further investigated in larger longitudinal interventional studies. In a recent longitudinal study, we were able to demonstrate that SoC and self-caring behavior in youth residential caregivers protect against burnout (40). The current findings extend the protective role of resilience onto psychophysiological alterations in hair samples of caregivers. Our findings have implications for promoting self-care behavior, as well as of implementing and cultivating a meaningful, comprehensible, and manageable professional climate in all facets of caregiving environments and in particular in the broader child welfare context. The least resilient, e.g., those individuals with low SoC were shown to be the most likely to increase their SoC level in an interventional study (38). Therefore, professional trainings of health promotion practices should focus on fostering resilience capacities in such individuals (91). Several different intervention programs exist to enhance resilience and were for example shown to be able to increase sense of coherence and quality of life (30, 92). Trauma-informed self-care, which includes seeking supervision, working within teams, balancing caseloads and developing a plan for work-life balance, was found to be protective against the development of burnout and secondary traumatic stress (25). Nevertheless, two recent systematic reviews concluded that self-care still takes a back seat in social work, and little is known about the efficacy of specific self-care practices (93, 94). Trauma-informed care (TIC) concepts that also focus on implementing resilience practices for caregivers might be a promising approach for reducing the emotional burden of employees and institutions and were shown to reduce hair cortisol levels of caregivers that were trained in TIC (95). However, more intervention research implementing psychophysiological assessments of stress that replicate our findings in prospective, longitudinal studies and the integration of such findings into educational programs is needed for improving resilience and maintaining empowered and healthy caregivers.

### Conclusion

This study is the first comprehensive investigation of multiple constructs related to resilience and their association to hair cortisol and DHEA levels in a sample of youth residential caregivers, a population prone to chronic stress. Sampling hair to measure cortisol and DHEA is a feasible, non-invasive approach to assess chronic stress, not only through questionnaires psychologically, but on a psychophysiological level. Our findings imply that youth residential care institutions might benefit from programs focusing on enhancing SoC and self-care practices. Cultivating a meaningful, comprehensible and manageable professional climate in caregiving environments and implementing self-care in routine practices might enhance not only well-being but also might improve physical health of professional caregivers and in this way buffer adverse health effects of chronic stressors.

## Data Availability Statement

The datasets presented in this article are not readily available because Data is only available upon request to the senior author and if shared will be protected by a signed agreement between the respective institutions that ensure ethical standards and legal requirements to be met. Requests to access the datasets should be directed to marc.schmid@upk.ch.

## Ethics Statement

The studies involving human participants were reviewed and approved by The leading Ethics Committee Basel-Stadt and Basel-Land (EKBB, Ref. Nr. 288/12), as well as the Cantonal Ethics Committee Bern (KEK-BE, Ref. Nr. 014/13), Ethics Committee St. Gallen (EKSG, Ref. Nr. 13/003), Ethics Committee Appenzell Ausserrhoden (EKAR, Ref. Nr. 34), Cantonal Ethics Committee Luzern (KEK-LU, Ref. Nr. 13009) and the Cantonal Ethics Committee Zürich (KEK-ZH, Ref. Nr. 2013-0030) approved the overall project. The patients/participants provided their written informed consent to participate in this study.

## Author Contributions

DB, NK, CB, and MSchm were major contributors in writing the manuscript and responsible for the interpretation of the findings. DB and NK performed all statistical analyses and wrote main parts of the manuscript. MSchm and JF organized funding for the study. MSchm was PI on the study and was a major contributor to the conceptualization of the manuscript. AE analyzed hair samples in her lab. AB was responsible for transcription and analyses of AAP interviews. NK, MSchr, VC, JF, AO'D, CB, and MSchm contributed to the interpretation and implications of the findings. All authors contributed to the article and approved the submitted version.

## Conflict of Interest

The authors declare that the research was conducted in the absence of any commercial or financial relationships that could be construed as a potential conflict of interest.
