# Supplementary material for: The Stress of Caring—Resilience and HPA-Axis Activity in Hair Samples of Youth Residential Caregivers
Source: Front Psychiatry. 2020 Dec 21;11:556486. doi: 10.3389/fpsyt.2020.556486 (PMC7779549; doi:10.3389/fpsyt.2020.556486)
Supplement: Supplementary file 1 [file Data_Sheet_1.docx]

| **Table S1.** | | | | | | | | | |
| --- | --- | --- | --- | --- | --- | --- | --- | --- | --- |
| **A** | **A1 RATIO** | | | **A2 CORT** | | | **A3 DHEA** | | |
|  | *β* | *CI* | *p* | *β* | *CI* | *p* | *β* | *CI* | *p* |
| Z-SoC | -0.36 | -0.54 – -0.19 | **<0.001** | -0.10 | -0.29 – 0.08 | 0.263 | 0.28 | 0.11 – 0.46 | **0.002** |
| Gender (F) | 0.19 | -0.15 – 0.54 | 0.269 | -0.19 | -0.56 – 0.18 | 0.318 | -0.46 | -0.81 – -0.11 | **0.010** |
| Age | -0.00 | -0.02 – 0.01 | 0.728 | -0.01 | -0.03 – 0.01 | 0.387 | 0.00 | -0.02 – 0.02 | 0.919 |
| N | 132 | | | 131 | | | 131 | | |
| R^2^ / R^2^ adj | 0.153 / 0.133 | | | 0.022 / -0.002 | | | 0.155 / 0.135 | | |

| **Table S2.** | | | | | | | | | |
| --- | --- | --- | --- | --- | --- | --- | --- | --- | --- |
| **B** | **B1 RATIO** | | **B2 CORT** | | | | **B3 DHEA** | | |
|  | *β* | *CI* | *p* | *β* | *CI* | *p* | *β* | *CI* | *p* |
| Z-SEF | -0.05 | -0.24 – 0.13 | 0.567 | 0.11 | -0.09 – 0.30 | 0.278 | 0.13 | -0.06 – 0.31 | 0.174 |
| Gender (F) | 0.37 | -0.00 – 0.74 | 0.051 | -0.17 | -0.54 – 0.21 | 0.379 | -0.58 | -0.94 – -0.22 | **0.002** |
| Age | -0.01 | -0.03 – 0.01 | 0.407 | -0.01 | -0.03 – 0.01 | 0.247 | 0.00 | -0.02 – 0.02 | 0.902 |
| N | 131 | | 130 | | | | 130 | | |
| R^2^ / R^2^ adj. | 0.049 / 0.027 | | 0.024 / 0.001 | | | | 0.112 / 0.091 | | |

| **Table S3.** | | | | | | | | | |
| --- | --- | --- | --- | --- | --- | --- | --- | --- | --- |
| **C** | **C1 RATIO** | | | **C2 CORT** | | | **C3 DHEA** | | |
|  | *β* | *CI* | *p* | *β* | *CI* | *p* | *β* | *CI* | *p* |
| Z-SEF | -0.24 | -0.41 – -0.07 | **0.005** | -0.10 | -0.27 – 0.07 | 0.262 | 0.21 | 0.04 – 0.38 | **0.016** |
| Gender (Fem.) | 0.36 | 0.01 – 0.71 | **0.043** | -0.22 | -0.58 – 0.15 | 0.241 | -0.62 | -0.96 – -0.27 | **0.001** |
| Age | -0.01 | -0.03 – 0.01 | 0.303 | -0.01 | -0.03 – 0.01 | 0.231 | 0.00 | -0.01 – 0.02 | 0.698 |
| N | 133 | | | 132 | | | 131 | | |
| R^2^ / R^2^ adj. | 0.092 / 0.071 | | | 0.031 / 0.008 | | | 0.118 / 0.092 | | |

**Supplementary Figure 1.** Associations between stress measures in hair samples and resilience measures from linear regression models including age and gender and an gender x resilience interaction term. *β* = standardized regression coefficients, * p<0.05 ** p<0.01 *** p<0.001.

Findings on Model A3

|  | **Log-DHEA** | | |
| --- | --- | --- | --- |
| *Predictors* | *Estimates* | *CI* | *p* |
| Intercept | 1.18 | 0.94 – 1.43 | **<0.001** |
| Z-SoC | -0.04 | -0.16 – 0.08 | 0.522 |
| Gender [Female] | -0.21 | -0.34 – -0.08 | **0.001** |
| Age | -0.00 | -0.01 – 0.01 | 0.805 |
| Z-SoC * Gender [Female] | 0.18 | 0.04 – 0.32 | **0.013** |
| N | 131 | | |
| R^2^ / R^2^ adjusted | 0.195 / 0.170 | | |
